# Annual Address to the Chester County Medical Society; Delivered April 25, 1848

**Published:** 1848-07

**Authors:** 


*Annual Address to the Chester County Medical Society; delivered April* 25, 1848, by Wilmer Worthington, M. D.

This is a sensible address, mainly devoted to the inculcation of sound ethics, in the course of which the author is necessarily led to speak of modern quackery, Thompsonism, Perkinsism, Homœopathy, Hydropathy, &c. Homœopathy, is properly examined, and its ridiculous pretensions rightly exposed. But that the subject has grown so stale as scarcely to attract notice any longer, we should be tempted to extract some of the passages in this address wherein the humbug is most effectually dissected.

